# Diversity of small molecule HIV‐1 latency reversing agents identified in low‐ and high‐throughput small molecule screens

**DOI:** 10.1002/med.21638

**Published:** 2019-10-13

**Authors:** Pargol Hashemi, Ivan Sadowski

**Affiliations:** ^1^ Biochemistry and Molecular Biology, Molecular Epigenetics, Life Sciences Institute University of British Columbia Vancouver British Columbia Canada

**Keywords:** chemical structures, HIV‐1, latency reversing agents, latent viral reservoirs, shock and kill, signal transduction, small molecule screens, structure‐activity relationship, transcriptional regulation

## Abstract

The latency phenomenon produced by human immunodeficiency virus (HIV‐1) prevents viral clearance by current therapies, and consequently development of a cure for HIV‐1 disease represents a formidable challenge. Research over the past decade has resulted in identification of small molecules that are capable of exposing HIV‐1 latent reservoirs, by reactivation of viral transcription, which is intended to render these infected cells sensitive to elimination by immune defense recognition or apoptosis. Molecules with this capability, known as latency‐reversing agents (LRAs) could lead to realization of proposed HIV‐1 cure strategies collectively termed “shock and kill,” which are intended to eliminate the latently infected population by forced reactivation of virus replication in combination with additional interventions that enhance killing by the immune system or virus‐mediated apoptosis. Here, we review efforts to discover novel LRAs via low‐ and high‐throughput small molecule screens, and summarize characteristics and biochemical properties of chemical structures with this activity. We expect this analysis will provide insight toward further research into optimized designs for new classes of more potent LRAs.

## 1 INTRODUCTION

The transcriptionally silent human immunodeficiency virus (HIV‐1) provirus produced in memory CD4^+^ T lymphocytes of infected patients represents a major impediment toward eradicating the virus, and infected patients are destined to remain on current therapies for the remainder of their lives to prevent virus rebound.^1^, ^2^, ^3^, ^4^ Latent viral reservoirs are unrecognizable by the immune system, and their presence is not affected by current antiretroviral therapies.^5^ Therefore, developing a cure for HIV/acquired immunodeficiency syndrome, and long‐term management of this disease will require strategies that target the latently infected cell population.^6^ Because latently infected cells are indistinguishable from uninfected cells, there is considerable interest in identifying biomarkers for this population; or, in the absence of such a suitable macromolecule, devising a means to expose these cells to enable clearance by the immune system, or additional designed therapies to force their elimination.^6^, ^7^ Toward this end, one possible strategy, of various that have been proposed,^8^, ^9^, ^10^, ^11^ may involve treatment with agents that induce viral gene expression in latent cellular reservoirs, which would expose this population via the appearance of intracellular and cell surface viral proteins.^12^, ^13^, ^14^ This may render these infected cells susceptible to a boosted immune response, viral‐induced apoptosis, or additional therapies specifically designed to eliminate HIV‐1‐infected cells.^13^, ^14^ A number of variations of this general strategy have been proposed and are commonly referred to as the “shock and kill” therapeutic approach.^13^ Central to the success of this strategy is the development of an effective means for reactivating HIV‐1 provirus expression in the latently infected population. Most efforts toward this goal have focused on identifying small molecule compounds with the capacity to reactivate transcription from the HIV‐1 5′ long terminal repeat (LTR) to a level that would enable production of sufficient amounts of the viral transactivator of transcription (TAT), which produces a strong positive feedback effect, thereby resulting in expression of viral proteins.^15^ Small molecules with this capacity are known as latency‐reversing agents (LRAs). Discovery of novel LRAs, and redirecting previously characterized bioactive molecules for this purpose have been the focus of many studies over the past decade.^16^

Initial clinical trials investigating feasibility of the shock and kill strategy, with aviremic patients on antiretroviral therapy (ART), have employed chromatin‐modifying agents, mostly histone deacetylase inhibitors (HDACIs), or cytokines that activate immune cell signaling.^14^ Results from these studies have revealed that successful implementation of this general approach will likely depend on several crucial factors relating to influence of the chromosomal integration site on viral transcriptional responses, and enhanced killing of cells where viral expression has become reactivated. Trials using HDACIs as LRAs have indicated that although viral replication can be induced in patients’ T cells, this treatment alone is not capable of reducing the latently infected population.^17^, ^18^, ^19^, ^20^ This limitation can likely be attributed to inadequate anti‐HIV‐1 cellular immune response in these patients, but also because of limitations of the HDACIs, which are only capable of inducing a subset of provirus integrations. Several studies have confirmed this limitation using cell lines with HIV‐1 reporter virus where it was shown that the site of chromosomal integration influences responsiveness to signaling agonists and chromatin‐modifying agents.^21^, ^22^, ^23^ Importantly, therefore, a critical property of LRAs is the capacity to induce a broad range of provirus integrated at diverse chromosomal locations. Consequently, identification of effective novel LRAs must involve screens that employ multiple different in vitro latency cell lines, in addition to infected normal CD4^+^ T cells, and latently infected cells from patients on ART. Additional important considerations include a requirement for minimal toxicity, and negligible effects on global T cell activation. Furthermore, there is increasing recognition that no single LRA is likely capable of inducing the full spectrum of latent provirus required to purge a sufficient fraction of infected cells, and therefore most recent efforts have focused on developing and characterizing combinations of compounds that produce synergistic effects on reactivation of individual provirus, and exert a broader effect on the latently infected population as a whole.^24^, ^25^, ^26^, ^27^, ^28^

Thus far, none of the LRAs examined in clinical trials have reduced the size of persistent HIV‐1 infection.^6^, ^29^, ^30^, ^31^, ^32^ Therefore, new classes and combinations of molecules with optimized pharmacological features must be developed to improve the shock parameter of the potential shock and kill strategy for therapeutic purposes. To this end, we review results of efforts to date toward discovering novel effective LRAs using low‐ and high‐throughput (HTP) screening of small molecule libraries. We compare structural properties and pharmacological features of the most effective compounds from these screens, and their capacity to produce synergistic responses with previously characterized LRAs. Comparison of novel molecules identified in these screens may reveal important chemical scaffolds, functional groups, and specific moieties that provide important features required for antilatency activity of particular classes of compounds. Therefore, we expect this analysis will aid in the design of optimized chemical structures for future research toward development of more potent LRAs for use in potential strategies to purge HIV‐1 infections.

## 2 LRAS IDENTIFIED FROM SMALL MOLECULE SCREENS EXHIBIT DIVERSE CHEMICAL STRUCTURES

We examined results from the 18 separate studies published to date (Table 1) involving screens of small molecule compound libraries for the capability to induce transcription of latent HIV‐1 reporter provirus. Most of these studies employed initial screens involving previously characterized cell line models for latency, most commonly the Jurkat T cell leukemia‐derived J‐LAT lines, where green fluorescent protein (GFP) expression is dependent on expression from the HIV‐1 5′ LTR. Several screens were performed with partially immortalized Bcl‐2‐transduced resting CD4^+^ T cells infected with a GFP‐expressing HIV‐1 reporter virus. Most screens have involved smaller libraries of compounds with known biological activities, natural compounds, or compounds with defined targets, such as epigenetic modifiers, and protein kinases or phosphatases. Five screens have involved libraries of synthetic chemical compounds with diverse structures (Table 1). As detailed above, compounds capable of inducing HIV‐1 provirus expression in initial screens, and which produce a dose‐dependent response in reanalysis, are typically re‐examined for this activity using additional reporter virus cell lines to determine whether the compound(s) are capable of reactivating a broad spectrum of HIV‐1 provirus integrants. Importantly, most studies also examine effectiveness for induction of replication‐competent virus from CD4^+^ T cells isolated from aviremic patients on ART, using a quantitative viral outgrowth assay, or induction of viral RNA by quantitative reverse transcription polymerase chain reaction (Table 1 and 2). Finally, the capacity of novel LRAs to produce synergistic induction of provirus expression in combination with previously characterized LRAs, typically signaling agonists or HDAC inhibitors, are often assessed. The efficacy of these novel compounds for induction of HIV‐1 transcription in reporter cell lines, reactivation of virus replication in primary cells from aviremic infected patients on ART, and their capacity to produce synergistic responses are summarized in Table 2.

**Table 1:** Overview of studies to identify novel latency reversing agents

Reference^a^	Library; initial # compounds	LRA(s)^b^	In vitro cell models^c^	TCA^d^
Savarino et al^33^	Institutional library of HDACIs; 32	MS‐275	ACH‐2, U1, J‐Lat A1	NI
Yang et al^34^	MicroSource Spectrum, JHDL; ~5000	5HN	Bcl‐2 transduced‐rCD4+ T cells, J‐Lat	N
Micheva‐Viteva et al^35^	Chemical compounds; 200 000	AV6	24STNLSG, ACH‐2, 19STNLSG, NLRlucRFP‐infected rCD4^+^ T cells	N
Xing et al^36^	MicroSource Spectrum, JHDL; ~500	Disulfiram	Bcl‐2 transduced‐rCD4+ T cells	N
Xing et al^37^	MicroSource Spectrum; 2000	Quinolin‐8‐ol	Bcl‐2 transduced‐rCD4+ T cells, J‐Lat	N
Gallastegui et al^38^	Chemical compounds; 6000	MMQO	J‐Lat A2, J‐Lat clones, J‐Lat pool, U1, ACH‐2	N
Shishido et al^24^	Chemical compounds, in‐house drugs/compounds; ~80 000	Aclacinomycin	J89GFP, CA5, EF7, HIV‐1 NL4‐3 infected CD8^+^ T cell‐depleted PBMCs	NI
Doyon et al^39^	Natural compounds; 640	57704	J89GFP, ACH‐2, U1	N
Tyagi et al^40^	Sulfonamide‐containing compounds; 38	SMAPP1	CEM T cells, Jkt‐pHR'P‐luc cells, THP1‐pHR'P‐luc cells, HIV pHR'P‐PNL‐H13LTat‐δNef‐GFP‐infected CD4^+^ T/PBMCs	NI
Stoszko et al^41^	Panel of BAF inhibitors; 18	Pyrimethamine; CAPE	J‐Lat clones (A2; 11.1), Bosque and Lassen cell models	N
Ao et al^42^	Chemical compounds, kinase inhibitors; >1500	PKC412	ACH‐2, TZMb‐1, HIV‐1 pNL‐4.3‐infected CD4^+^ T cells	NI
Bosque et al^43^	Natural compounds; 2000	HODHBt	Primary T_CM_ model, J‐Lat clones	N
Kobayashi et al^44^	Epigenetic modulating compounds; 94	Chidamide; MS‐275	gGn‐p* infected rCD4^+^ T cells, 24STNLEG	NI
Albert et al^27^	Panel of isoform‐targeted HDACIs; 15	Largazole	J‐Lat 10.6, Greene cell model	N
Wang et al^45^	Natural compounds; ~100	EK‐16A	C11 Jkt, J‐Lat clones (10.6; 6.3)	N
Hashemi et al^28^	Chemical compounds; ~180 000	PH01‐PH05	J‐Lat A1, Jkt^Tat^ LTR‐Luc, Jkt^Tat^ LTR‐DsRed clones (11; 131)	N
Gohda et al^46^	Chemical kinase inhibitors; 378	BI‐2536; BI‐6727	THP‐1‐NanoLuc Clones (95; 225), ACH‐2, U1	NI
Richard et al^47^	Natural compounds; 257	Psammaplin A; aplysiatoxin; debromoaplysiatoxin	J‐Lat clones (9.2, 8.4, 10.6)	NI

Abbreviations: 5HN, 5‐hydroxynaphthalene‐1,4‐dion; BAF, BRG1/BRM‐associated factor; CAPE, caffeic acid phenethyl esther; GFP, green fluorescent protein; HDACI, histone deacetylase inhibitor; HODHBt, 3‐hydroxy‐1,2,3‐benzotriazin‐4(3*H*)‐one; JHDL, Johns Hopkins Drug Library; Jkt, Jurkat; LRA, latency reversing agent; LRA, latency reversing agent; MMQO, 8‐methoxy‐6‐methylquinolin‐4‐ol; N, no; NI, not investigated; rCD4^+^ T cells, resting CD4^+^ T cells; SMAPP1, small molecule activator of protein phosphatase 1; TCA, T cell activation.

^a^ Study reference.

^b^ Novel LRA(s) identified.

^c^ In vitro HIV‐1 latency cellular models utilized.

^d^ Compound(s) induce T cell activation.

**Table 2:** Efficacy of LRAs examined in in vitro and ex vivo studies

LRA(s)	Cell line^a^	Time (h)^b^	EC (µM)^c^	Synergy^d^	Patient^e^	C, µM (time)^f^	Technique^g^
MS‐275^33^	ACH2	72	0.10	BSO	NI		
5HN^34^	Bcl‐2 transduced‐rCD4^+^ T cells	40‐48	0.5	NI	NI		
AV6^35^	24STNLSG	48	12	VA	NI		
Disulfiram^36^	Bcl‐2 transduced‐rCD4^+^ T cells	24/48	0.3‐0.5, 5‐10	NI	NI		
MMQO^38^	J‐Lat	24	80	TNF‐α, PMA, TSA	PBMCs	80 (36 h)	RT‐qPCR
Aclacinomycin^24^	CA5 cells	24	0.5	TNF‐α	NI		
57704^39^	U1	24	5‐9	Prostratin, SAHA, DSF, BIX‐01294	CD8^+^‐depleted MNCs	1/5 (7 d)	RT‐qPCR
SMAPP1^40^	CEM T cells	24	10	NI	NI		
Pyrimethamine; CAPE^41^	J‐Lat 11.1	24	2	NI	CD4^+^ T cells	20/1 (12 h)	RT‐qPCR
PKC412^42^	ACH2	48	0.5	VOR	NI		
HODHBt^43^	Primary T_CM_ model	24	100	NI	rCD4^+^ T cells	100 + IL‐2 (24 h)	VOA, ELISA
Chidamide; MS‐275^44^	gGn‐p* infected rCD4^+^ T cells	48	2, 20	NI	NI		
Largazole^27^	J‐Lat 10.6	24	0.15	Bryostatin, SUW133, SUW124	NI		
EK‐16A^45^	J‐Lat 10.6	28	4.06	5‐Aza, JQ1, I‐Bet151, Romidepsin, SAHA	rCD4^+^ T cells	0.05 (18 h)	
PH01‐PH05^28^	Jurkat^Tat^ LTR‐Luciferase cells	24	0.1‐ 5.9	SAHA, chaetocin, ionomycin, PEP005	CD4^+^ T cells	1 (24 h)	VOA, RT‐qPCR
BI‐2536; BI‐6727^46^	ACH2/U1	16	1	SAHA, prostratin	PBMCs	1 (16)	RT‐qPCR
Psammaplin A; aplysiatoxin; debromoaplysiatoxin^47^	J‐Lat 9.2	24	1.9	TNF‐α, prostratin	PBMCs	3.8 (24 h)	ELISA
				TNF‐α, panobinostat		1.1 (24 h)	
				TNF‐α, panobinostat		1.3 (24 h)	

Abbreviations: 5‐Aza, 5‐azacytidine; 5HN, 5‐hydroxynaphthalene‐1,4‐dion; BSO, buthionine sulfoximine; C, concentration; CAPE, caffeic acid phenethyl esther; DSF, disulfiram; EC, effective concertation; ELISA, enzyme‐linked immunosorbent assay; HIV, human immunodeficiency virus; HODHBt, 3‐hydroxy‐1,2,3‐benzotriazin‐4(3*H*)‐one; IL, interleukin; LRA, latency reversing agent; MMQO, 8‐methoxy‐6‐methylquinolin‐4‐ol; MNC, mononuclear cell; NI, not investigated; PBMC, peripheral blood mononuclear cell; PMA, phorbol 12 myristate 13‐acetate; RT‐qPCR, reverse transcription‐quantitative polymerase chain reaction; SAHA, suberoylanilide hydroxamic acid; SMAPP1, small molecule activator of protein phosphatase 1; TNF‐α, tumor necrosis factor α; TSA, trichostatin A; VA, valproic acid; VOA, viral outgrowth assay; VOR, vorinostat.

^a^ HIV‐1 reporter cell line utilized for dose‐response study.

^b^ Treatment time (h).

^c^ Effective concentration for induction of HIV expression.

^d^ Treatment(s) found to produce synergistic HIV expression response in combination with the LRA.

^e^ Effect of LRA for reactivation of HIV from cells purified from aviremic patients on antiretroviral therapy.

^f^ Concentration of LRA examined on primary cells for the indicated time period.

^g^ Technique for measuring viral production (viral mRNA/p24) posttreatment.

**Table 3:** Summary of small molecule LRAs identified in screens

LRA^a^	IUPAC designation^b^	MW^c^	Target(s)^d^	Reference(s)^e^
*Group 1: Epigenetic modifiers*				
MS‐275	Pyridin‐3‐ylmethyl *N*‐[[4‐[(2‐aminophenyl)carbamoyl]phenyl]methyl]carbamate	376.41	Class I HDACs (HDAC1, 2, 3)	33, 48, 49, 50, 51
Chidamide	*N*‐(2‐Amino‐5‐fluorophenyl)‐4‐[[[(*E*)‐3‐pyridin‐3‐ylprop‐2‐enoyl]amino]methyl]benzamide	390.42	HDAC 1, 2, 3, and 10	44, 52, 53, 54
Largazole	*S*‐{(3*E*)‐4‐[(5*R*,8*S*,11*S*)‐8‐Isopropyl‐5‐methyl‐6,9,13‐trioxo‐10‐oxa‐3,17‐dithia‐7,14,19,20‐tetraazatricyclo[14.2.1.1~2,5~]icosa‐1(18),2(20),16(19)‐trien‐11‐yl]‐3‐buten‐1‐yl} octanethioate	622.85	Class I HDACs (HDAC 1, 2, 3, and 8)	27, 55, 56
Psammaplin A	(2*E*)‐3‐(3‐Bromo‐4‐hydroxyphenyl)‐*N*‐[2‐[2‐[[(2*E*)‐3‐(3‐bromo‐4‐hydroxyphenyl)‐2‐hydroxyiminopropanoyl]amino]ethyldisulfanyl]ethyl]‐2‐hydroxyiminopropanamide	664.38	Class I HDACs (HDAC 1)	47, 57, 58
MMQO	8‐Methoxy‐6‐ methylquinolin‐4‐ol	189.21	BRD4	38, 59
BI‐2536	4‐[[(7*R*)‐8‐Cyclopentyl‐7‐ethyl‐5‐methyl‐6‐oxo‐7*H*‐pteridin‐2‐yl]amino]‐3‐methoxy‐*N*‐(1‐methylpiperidin‐4‐yl)benzamide	521.666	PLK1, PLK2, PLK3, BRD4	46, 60, 61, 62
BI‐6727	*N*‐[4‐[4‐(Cyclopropylmethyl)piperazin‐1‐yl]cyclohexyl]‐4‐[[(7*R*)‐7‐ethyl‐5‐methyl‐6‐oxo‐8‐propan‐2‐yl‐7*H*‐pteridin‐2‐yl]amino]‐3‐methoxybenzamide	618.827	PLK1, PLK2, PLK3, BRD4	46, 61, 63
*Group 2: Chromatin modulators*				
Pyrimethamine	5‐(4‐Chlorophenyl)‐6‐ethylpyrimidine‐2,4‐diamine	248.71	DHFR, BAF complex	41, 64
CAPE	2‐Phenylethyl (*E*)‐3‐(3,4‐dihydroxyphenyl)prop‐2‐enoate	284.31	HIV‐1 integrase, NFκB signaling pathway, BAF complex	41, 65, 66, 67, 68
*Group 3: Signaling effectors/modulators*				
5HN	5‐Hydroxynaphthalene‐1,4‐dione	174.15	Parvulin PPIase family members (Pin1), HIV‐RT, NFκB signaling pathway	34, 69, 70, 71
AV6	4‐3,4‐Dichloroanilino‐6‐methoxyquinoline	333.05	NFAT‐signaling pathway	35
Disulfiram	Diethylcarbamothioylsulfanyl *N*,*N*‐diethylcarbamodithioate	296.54	ALDH, DβH, Akt‐signaling (PTEN)	72, 73, 74, 75
57704	1,2,9,10‐Tetramethoxy‐7*H* dibenzo[de,g]quinolin‐7‐one	351.35	Akt‐signaling pathway (PI3K p110)	39
PKC412	*N*‐((9*S*,10*R*,11*R*,13*R*)‐10‐Methoxy‐9‐methyl‐1‐oxo‐2,3,10,11,12,13‐hexahydro‐9,13‐epoxy‐1*H*,9*H*‐diindolo(1,2,3‐GH:3′,2′,1′‐lm)pyrrolo(3,4‐j)(1,7)benzodiazonin‐11‐yl)‐*n*‐methylbenzamide	570.6	PKC, FLT3, ZNF198‐FGFR1, PDGFR‐, c‐Kit receptor, VEGF‐R2, JNK pathway, NFκB signaling pathway	42, 76, 77, 78, 79
HODHBt	3‐Hydroxy‐1,2,3‐benzotriazin‐4(3*H*)‐one	163.13	STAT5 SUMOylation	43
EK‐16A	[(1*S*,4*S*,5*S*,6*R*,9*S*,10*R*,12*R*,14*R*)‐5,6‐Dihydroxy‐7‐(hydroxymethyl)‐3,11,11,14‐tetramethyl‐15‐oxo‐4‐tetracyclo[7.5.1.0.1,5.010,12]pentadeca‐2,7‐dienyl] (*Z*)‐2‐methylbut‐2‐enoate	658.89	PKC (PKC‐NFκB signaling pathway, pTEFb	45
Aplysiatoxin	(1*S*,3*R*,4*S*,5*S*,9*R*,13*S*,14*R*)‐3‐[(2*S*,5*S*)‐5‐(2‐Bromo‐5‐hydroxyphenyl)‐5‐methoxy‐2‐pentanyl]‐13‐hydroxy‐9‐[(1*R*)‐1‐hydroxyethyl]‐4,14,16,16‐tetramethyl‐2,6,10,17‐tetraoxatricyclo[11.3.1.11,5]octadecane‐7,11‐dione	671.62	PKC signaling pathway	80, 81
Debromoaplysiatoxin	(1*S*,3*R*,4*S*,5*S*,9*R*,13*S*,14*R*)‐13‐Hydroxy‐9‐[(1*R*)‐1‐hydroxyethyl]‐3‐[(2*S*,5*S*)‐5‐(3‐hydroxyphenyl)‐5‐methoxypentan‐2‐yl]‐4,14,16,16‐tetramethyl‐2,6,10,17‐tetraoxatricyclo[11.3.1.11,5]octadecane‐7,11‐dione	592.72	PKC signaling pathway	80, 81
*Group 4: Transcriptional elongation modulators*				
Aclacinomycin	Methyl (1*R*,2*R*,4*S*)‐4‐[(2*R*,4*S*,5*S*,6*S*)‐4‐(dimethylamino)‐5‐[(2*S*,4*S*,5*S*,6*S*)‐4‐hydroxy‐6‐methyl‐5‐[(2*R*,6*S*)‐6‐methyl‐5‐oxooxan‐2‐yl]oxyoxan‐2‐yl]oxy‐6‐methyloxan‐2‐yl]oxy‐2‐ethyl‐ 2,5,7‐trihydroxy‐6,11‐dioxo‐3,4‐dihydro‐1*H*‐tetracene‐1‐carboxylate	811.87	Topoisomerase type 1 and 2, 20S proteasome (MPC), pTEFb	24, 82, 83
SMAPP1	1,2,3,4‐Tetrahydro‐2‐[(4 methylphenyl)sulfonyl]‐*N*‐[4‐[(2‐pyrimidinylamino)sulfonyl]phenyl]‐3‐isoquinolinecarboxamide	467.12	PP1	40

Abbreviations: 5HN, 5‐hydroxynaphthalene‐1,4‐dion; ALDH, aldehyde dehydrogenase; BAF, BRG1/BRM‐associated factor; BRD, bromodomain protein; Brd4, bromodomain‐containing protein 4; CAPE, caffeic acid phenethyl esther; DβH, dopamine β‐hydroxylase; DHFR, dihydrofolate reductase; FLT3, FMS‐like tyrosine kinase‐3; HDAC, histone deacetylase; HIV‐RT, human immunodeficiency virus reverse transcriptase; HODHBt, 3‐hydroxy‐1,2,3‐benzotriazin‐4(3*H*)‐one; JNK, c‐Jun N‐terminal kinases; LRA, latency reversing agent; MMQO, 8‐methoxy‐6‐methylquinolin‐4‐ol; MPC, multicatalytic proteinase complex; MW, molecular weight; NFκB, nuclear factor κ‐light‐chain‐enhancer of activated B cells; NFAT, nuclear factor of activated T‐cells; PDGF‐α, platelet‐derived growth factor‐α; PI3K, phosphoinositide 3‐kinase; Pin 1, peptidylprolyl cis/trans isomerase, NIMA‐interacting 1; PKC, protein kinase C; PLK, polo‐like kinase; PP1, protein phosphatase 1; PPIase, peptidylprolyl isomerase; pTEFb, positive transcription elongation factor b; PTEN, phosphatase and tensin homolog; SMAPP1, small molecule activator of protein phosphatase 1; STAT5, signal transducer and activator of transcription 5; VEGF‐R2, vascular endothelial growth factor receptor 2; ZNF198‐FGFR1, zinc finger 198‐FGF receptor 1 fusion tyrosine kinase.

^a^ Latency reversing agent, common designation.

^b^ International Union of Pure and Applied Chemistry designation.

^c^ Molecular weight (g/mol).

^d^ Known or suspected biological target(s).

^e^ References for screen and other compound's related studies.

**Figure 1:**
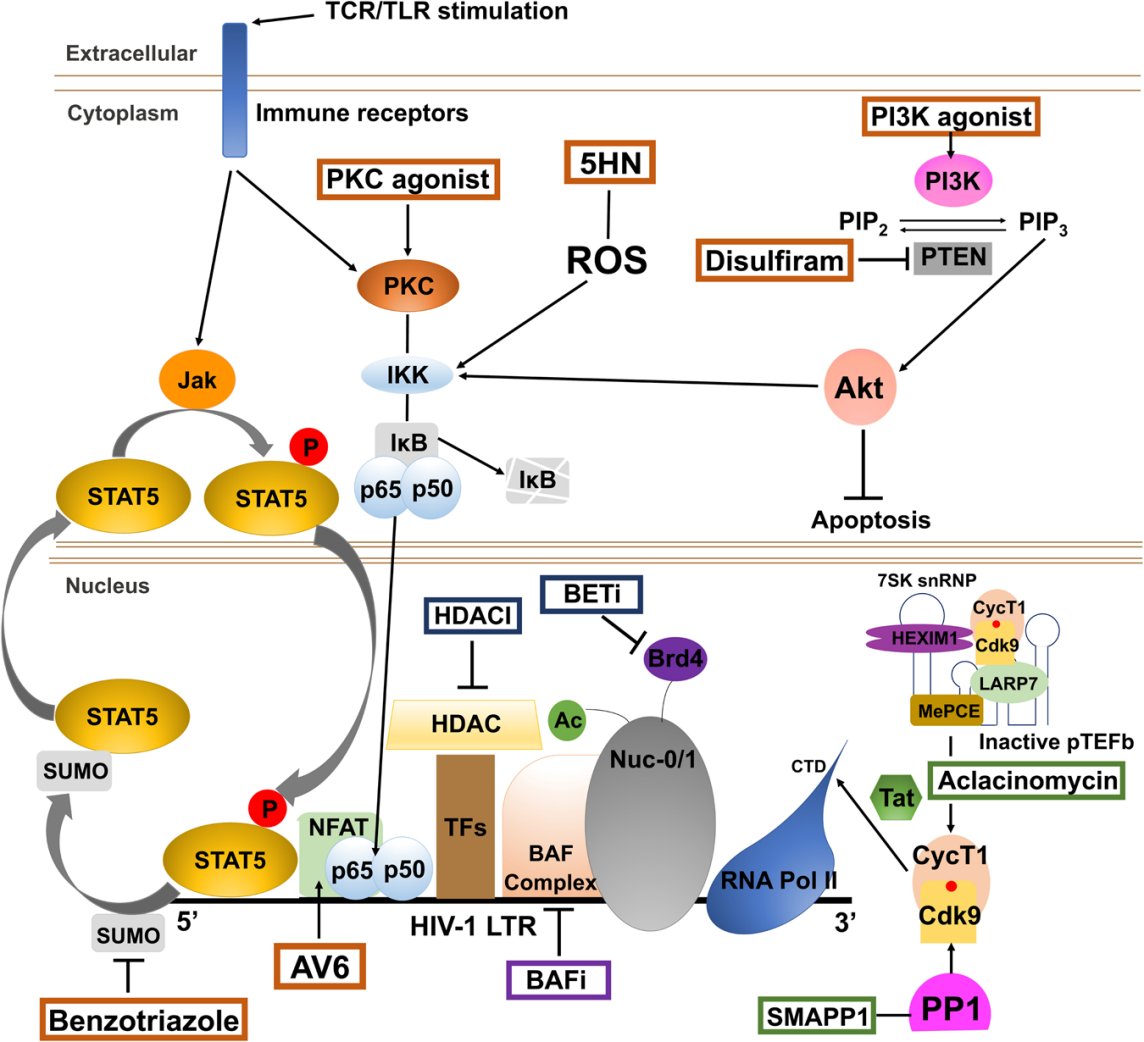
Latency reversing agents induce HIV‐1 provirus replication by diverse mechanisms. Small molecule compounds identified as LRAs cause reactivation of HIV‐1 transcription/replication through at least four distinct mechanisms, organized into Groups (Table 3), including: epigenetic modifiers (boxed in blue); chromatin modulators (purple); signaling effectors/modulators (orange); and RNA Pol II transcriptional elongation modulators (green). Epigenetic modifiers (blue), include HDAC and BET inhibitors that enhance viral transcription by modulating posttranslational modifications of core histone N‐terminal tails. Chromatin modulators (purple), include BAF inhibitors that regulate nucleosome positioning on the HIV‐1 5′ LTR (Nuc 0/1) and specifically disrupt Nuc 1 to allow activated transcription by RNA Polymerase II. Signaling effectors/modulators (orange) affect PI3K‐Akt, PKC‐NFκB, and Jak‐STAT5 pathways. Agonists activating PKC stimulate the transcription factor NFκB through activation of IKK and degradation of the inhibitor of NFκB (IκB). Signaling modulator 5HN also induces HIV‐1 expression through ROS‐stimulated IKK, and consequently NFκB activation. PI3K agonists directly activate PI3K, while disulfiram induces activation through this pathway by inhibiting the negative regulator PTEN, which also regulates IKK through the function of Akt. The JAK‐STAT pathway stimulates HIV expression through STAT5; benzotriazoles indirectly activate this effect by impairing STAT5 SUMOylation causing its retention in the nucleus. Signaling effector AV6 stimulates viral transcription by enhancing NFAT activity. Transcriptional elongation modulators (green) include SMAPP1, which enhances viral gene expression by activating Cdk9 of pTEFb, while aclacinomycin causes the dissociation of pTEFb from the inhibitory 7S snRNP complex. 5HN, 5‐hydroxynaphthalene‐1,4‐dion; AV6, antiviral 6; BAF, BRG1/BRM‐associated factor; BAFi, BAF complex inhibitor; BET, bromodomain and extra‐terminal; BETi, BET protein inhibitors; Brd4, bromodomain containing 4; CTD, C‐terminal domain; CycT1, cyclinT1; HADC, histone deacetylase; HDACI, histone deacetylase inhibitors; HEXIM1, hexamethylene bisacetamide inducible protein 1; HIV, human immunodeficiency virus; IKK, IκB kinase; IκB, inhibitor of κB; JAK, Janus kinase; LARP7, La ribonucleoprotein domain family member 7; LTR, long terminal repeat; MePCE, methylphosphate capping enzyme; NFAT, nuclear factor of activated T‐cells; NFκB, nuclear factor κ‐light‐chain‐enhancer of activated B cells; Nuc‐0/1, nucleosome‐0/1; PI3K, phosphoinositide 3‐kinases; PIP2, phosphatidylinositol (4,5)‐bisphosphate; PIP3, phosphatidylinositol (3,4,5)‐trisphosphate; PKC, protein kinase C; PP1, protein phosphatase 1; pTEFb, positive transcription elongation factor b; PTEN, phosphatase and tensin homolog; RNA Pol II, RNA polymerase II; ROS, reactive oxygen species; SMAPP1, small molecule activator of protein phosphatase 1; snRNP, small nuclear ribonucleoprotein; STAT5, signal transducer and activator of transcription 5; TCR, T cell receptor; TF, transcription factor; TLR, toll‐like receptor [Color figure can beviewed at wileyonlinelibrary.com]

To summarize effects of LRAs identified and characterized in these screens, we have classified the compounds into four groups, that include epigenetic modifiers, chromatin modulators, signaling effectors or modulators, and transcriptional elongation modulators (Table 3). Precise identification of cellular targets affected by previously uncharacterized compounds and, consequently, the molecular pathway(s) involved, is challenging and sometimes can remain elusive for decades. Furthermore, identification of molecular targets also requires detailed analysis of off‐target and secondary effects that may influence HIV‐1 transcription. Therefore, the groupings indicated here are intended to represent major pathways in which the compounds are most likely involved (Figure 1), but in many cases details of molecular effects have not been completely elucidated.

### 2.1 Group 1: Epigenetic modifiers

#### 2.1.1 MS‐275/entinostat, chidamide/Epidaza, largazole, and psammaplin

HDACIs have attracted most initial focus as LRAs in clinical trials, because several compounds with this activity had previously been employed as mood stabilizers and antiepileptics; the compounds have also been used in clinical trials for treatment of severe depression,^84^, ^85^ as well as various cancers.^86^, ^87^, ^88^ Similarly, several groups observed that HDACIs could elevate the expression of HIV‐1 provirus in cell line models of latency, and these observations led to clinical trials with patients on ART, with the goal of purging latently infected cells via “shock and kill.”^88^, ^89^ Structurally, HDACIs are classified into four main groups: hydroxamates, cyclic peptides, aliphatic acids, and benzamides.^88^, ^89^ Although their full mechanism of action needs elucidation, these compounds cause accumulation of acetylated histones and nonhistone proteins involved in the regulation of gene expression, cell proliferation, differentiation, and cell death.^88^, ^89^

**Figure 2:**
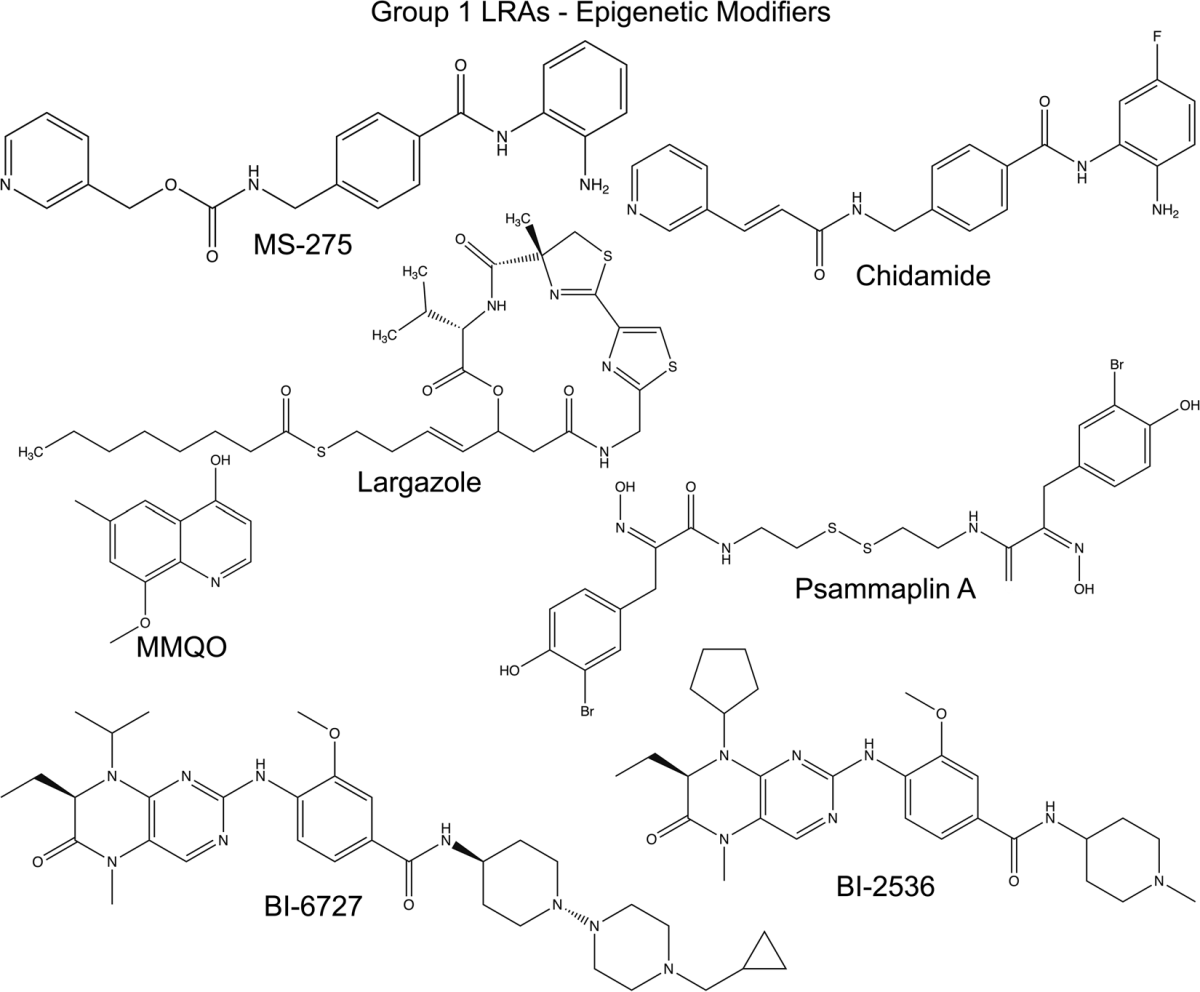
Chemical structures of group 1 LRAs, epigenetic modifiers. Shown are the chemical structures small molecule compounds identified as LRAs causing alteration of histone modifications. MS‐275, chidamide, Larazole, and psammaplin A were determined to inhibit class I HDACs. MMQO, BI‐2536, and BI‐6727 were identified as BET inhibitors. BET, bromodomain and extra‐terminal; HDAC, histone deacetylase; LRA,latency‐reversing agent; MMQO, 8‐methoxy‐6‐methylquinolin‐4‐ol

Eighteen human HDACs have been identified to date, which are organized into four classes based on structural identity with their yeast orthologues.^88^ The class I HDACs, primarily localized to the nucleus include HDAC 1, 2, 3, and 8, and are ubiquitously expressed in mammalian cell lines and tissues. HDACs in classes I, II, and IV share common features, including dependence on Zn^2+^ for enzymatic activity, while class III HDAC proteins have catalytic activity that requires a nicotinamide adenine dinucleotide (NAD+) cofactor.^88^ Class I HDACs play an important role for establishment of HIV‐1 latency in resting CD4^+^ T cells.^14^ These proteins are recruited to the HIV‐1 promoter by multiple transcription factors acting as transcriptional repressors in unstimulated cells, including yin yang 1, Ras‐responsive element binding factor 2 which is a protein complex made up of proteins transcription factor II‐I, and upstream‐stimulatory factor 1/2, nuclear factor κ‐light‐chain‐enhancer of activated B cells (NFκB) p50, and specificity protein 1, which cause repression of transcription.^14^ Therefore, the identification and/or development of potent inhibitors which specifically target these enzymes would be beneficial for disruption of HIV‐1 latency (HDACI; Figure 1).^90^ Toward this goal, the compound MS‐275 (Figure 2 and Table 3) was found to be a potent LRA, identified by screening a panel of HDACIs consisting of both nonclass and class‐specific HDACIs using in vitro latency cell models (Tables 1 and 2).^33^ This compound is a benzamide derivative, has affinity for class I HDACs and is particularly selective for HDAC 1 relative to HDAC 3 or HDAC 8 (Table 3). MS‐275 was previously characterized as an antitumor agent in several cancer‐related clinical studies as well as for the treatment of psychiatric disorders, and was shown to selectively cause elevated histone H3‐acetylation in a mouse brain model.^86^, ^91^ Structure‐activity relationship (SAR) studies examining potency of HDACIs for reversing HIV‐1 latency revealed essential features for this class of LRAs which include a cap group, polar connection unit, and a hydrophobic spacer (HS) with a hydroxamate or benzamide Zn^2+^ binding group coupled to the HS.^33^ Similar requirements for benzamide‐containing HDACIs, including MS‐275 and chidamide (Figure 2 and Table 3) were identified for latency reversing activity among epigenetic modifiers examined using a primary cell model of HIV‐1 latency (Tables 1 and 2).^44^ Latency reversing activity of MS‐275 was examined in combination with other molecules including buthionine sulfoximine, a glutathione‐synthesis inhibitor; prostratin, a nontumor‐promoting phorbol ester; and 5‐aza‐2′deoxycytidine, a DNA demethylating agent,^33^, ^92^, ^93^ and these studies reported various levels of potency, which is likely the result of differences in HIV‐1 reporter virus at different chromosomal regions. The related compound chidamide (Figure 2), showed better cell tolerance than the commonly used broad‐spectrum HDAC inhibitor suberoylanilide hydroxamic acid (SAHA), and was also able to disrupt viral silencing in primary CD4^+^ T cells isolated from patients.^52^, ^53^

A number of additional HDACIs with latency reversing activity have emerged from screens involving compounds from natural sources, including largazole and psammaplin A (Figure 2 and Table 3), which were identified in screens of class‐specific HDACIs, and chemical libraries from marine invertebrates and microorganisms (Tables 1 and 2).^27^, ^47^ Largazole (Figure 2), originally isolated from marine cyanobacteria, is a well‐known antiproliferation agent with specificity toward class I HDAC enzymes,^55^ and showed significant synergy for HIV‐1 provirus reactivation with analogs of bryostatin‐1, and produced minimal toxicity, when examined on J‐Lat reporter cell lines and resting CD4^+^ T cells.^27^ Psammaplin (Figure 2), which is also a class I HDAC inhibitor with antitumor activity was identified in a screen for LRAs from a library of natural compounds derived from marine invertebrates and microorganisms (Tables 1 and 2).^47^ These recently identified LRAs, largazole and psammaplin A, are structurally divergent from previously characterized HDACIs in that they possess a thioester linkage and intramolecular disulfide bond, respectively^55^, ^57^, ^94^ (Figure 2). Largazole is rapidly hydrolyzed to a thiol in the presence of plasma and active cellular proteins.^94^ In contrast, HDACIs with an intramolecular disulfide bond, like psammaplin A, are susceptible to reduction after cell uptake, leading to the production of monomers with thiol groups.^91^ The presence of a thiol group, in the hydrolyzed form of largazole and in the disulfide‐reduced form of psammaplin A, is essential for their inhibitory activity against HDAC molecules, as they chelate zinc ions present at the active site, which inhibits enzymatic activity.^90^, ^91^, ^95^

#### 2.1.2 8‐Methoxy‐6‐methylquinolin‐4‐ol, BI‐2536, and BI‐6727/volasertib

Nuclear bromodomain and extra‐terminal (BET) family proteins (bromodomain protein 2 (BRD2, BRD3, BRD4, and mBRDT), which recognize the acetylated N‐terminal tail of histones through their bromodomains (BDs), act as readers of acetylated lysine and are involved in regulating gene expression.^96^ They serve as recruitment platforms for transcriptional regulatory factors as well as chromatin re‐modelers. Generally, these proteins contain two tandem BDs, BD1 and BD2, in addition to a C‐terminal extra‐terminal domain. BET proteins have attracted recent attention as therapeutic targets in various disorders, such as cancer, inflammation, neurological diseases, and HIV‐1 persistent infection.^96^ Computational analysis of compounds with structural similarity to known BET‐inhibitor LRAs, and analysis using the J‐Lat A2 cell line, identified 8‐methoxy‐6‐methylquinolin‐4‐ol (MMQO) as a novel LRA (Tables 1 and 2),^38^ which was demonstrated to disrupt HIV‐1 latency via inhibition of the BET protein BRD4 (Figure 1, BETi; Figure 2; and Table 3).^59^ MMQO directly binds the BRD4 BD1 domain, where the aromatic ring with two methyl groups (ring A) intercalates into the BD1 acetyl‐lysine binding pocket, while the quinoline pyridine ring of MMQO is exposed outside of the BD1 binding pocket.^59^

Several additional BRD4/BET inhibitors have been identified as LRAs, namely BI‐2536 and BI‐6727 (Figure 1, BETi; Figure 2; and Table 3).^46^ Both of these compounds have pteridine groups (Figure 2), and were identified in screens of protein kinase inhibitors using latently infected monocytic THP‐1 cells (Tables 1 and 2).^46^ BI‐2536 was initially identified as an inhibitor of polo‐like kinase 1 (PLK1),^97^ which has a key regulatory function for mitotic progression.^60^ More recently, BI‐2536 was also shown to bind BDs, and structural analyses revealed specific interactions of BI‐2536 with the BRD4 hydrophobic cavity.^61^ The latency reversing activity of BI‐2536 and BI‐6727 are likely associated with inhibition of BRD4, rather than an inhibitory effect on protein kinases^46^; accordingly, other PLK inhibitors were unable to cause significant activation of HIV‐1 transcription in the same HIV‐1 reporter cell lines utilized for the initial screen.^46^ Furthermore, ChIP analyses indicates that treatment with either BI compound causes reduction in BRD4 recruitment to the HIV‐1 promoter,^46^ similar to the effect of JQ1, a previously identified LRA with known BRD4 inhibitory function.^98^, ^99^

### 2.2 Group 2: Chromatin modulators

#### 2.2.1 Pyrimethamine/Daraprim and caffeic acid phenethyl esther

**Figure 3:**
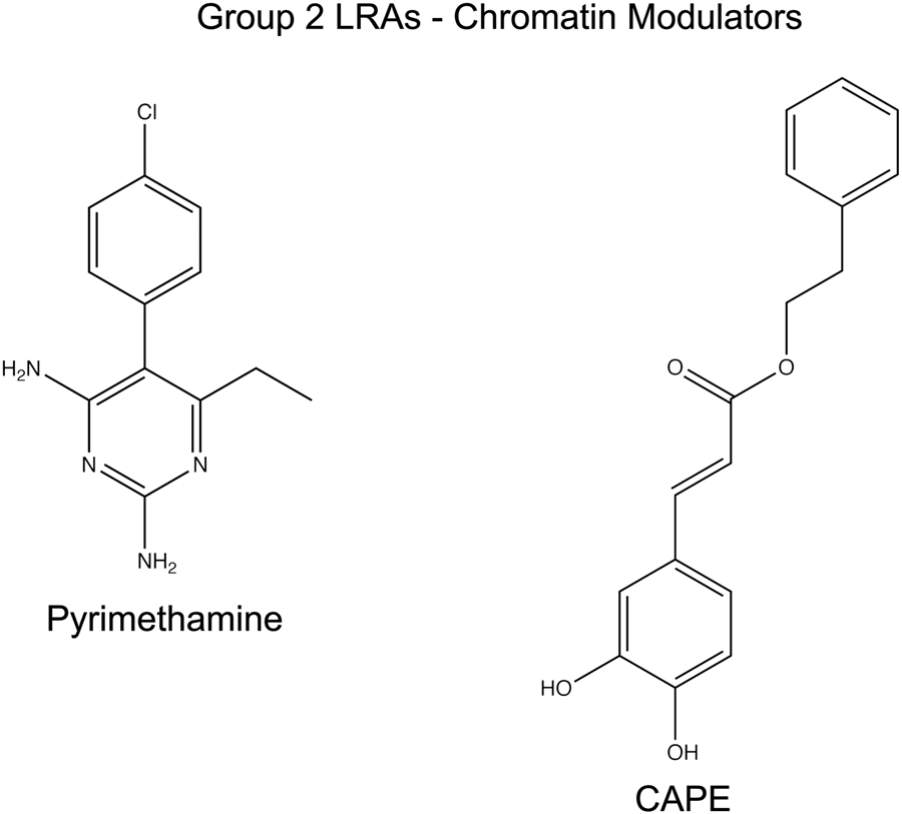
Chemical structures of group 2 LRAs, chromatin modulators. Shown are the structures of pyrimethamine and CAPE, that cause reactivation of HIV transcription through inhibition of the chromatin remodeling complex BAF. BAF, BRG1/BRM‐associated factors; CAPE, caffeic acid phenethyl esther; HIV, human immunodeficiency virus; LRA, latency‐reversing agent

The HIV‐1 LTR is known to have two strongly positioned nucleosomes, Nuc‐0 and Nuc‐1, flanking conserved elements within the viral 5′ LTR enhancer region.^100^, ^101^ Nuc‐1 is positioned immediately downstream of the transcriptional start site, and is restrictive to HIV‐1 transcription^102^, ^103^ (Figure 1, Nuc 0/1). Upon T cell activation, Nuc‐1 is disrupted through the function of nucleosome remodelers, such as the SWI/SNF (SWItch/sucrose nonfermentable) family member, polybromo‐associated BRG1/BRM‐associated factors (BAF), which enables activation of gene expression.^103^ Importantly, the adenosine triphosphate‐dependent BAF chromatin‐remodeling complex plays a vital role in establishing and maintaining viral latency by actively positioning Nuc‐1 near the transcription start site,^104^, ^105^ which implicates the BAF chromatin remodeler as a putative target for disruption of silencing. Accordingly, several BAF inhibitors were shown to reactivate viral transcription (Figure 1, BAFi),^41^, ^106^ including pyrimethamine and caffeic acid phenethyl esther (CAPE), in the J‐Lat reporter cell lines and in CD4^+^ T cells from HIV‐1‐infected patients (Figure 3, Tables 1 and 3). The effect of these compounds for reactivation of latency is mediated through inhibition of BAF250a which causes displacement of the complex from the viral promoter.^41^ Among these, pyrimethamine has antiprotozoan activity, and is FDA‐approved for treatment of HIV‐1‐infected patients prone to opportunistic infections.^107^ An unexpected finding was that the BAF inhibitor CAPE,^41^ a bioactive compound isolated from honey bee propolis, also inhibits activation of NFκB at relatively high concentrations,^65^, ^66^, ^67^ and at lower concentrations causes reactivation of latent provirus, but not NFκB p65 inhibition.^41^ Furthermore, both of these BAF inhibitors were also shown to be capable of preventing establishment of latent proviruses; Jurkat cells pretreated with these compounds produce decreased numbers of latently infected cells.^41^

### 2.3 Group 3: Signaling effectors/modulators

#### 2.3.1 5HN/juglone

**Figure 4:**
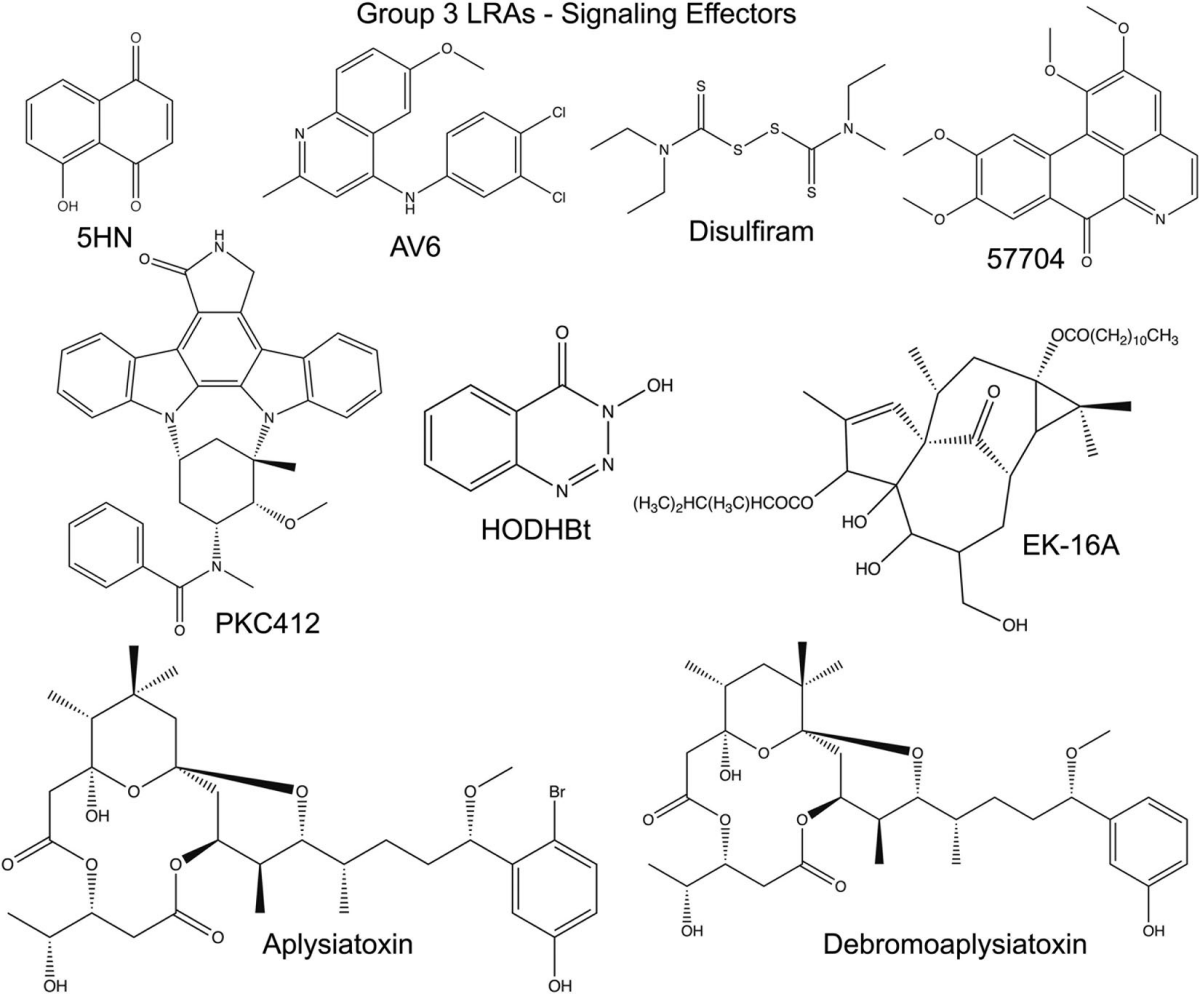
Chemical structures of group 3 LRAs, signaling effectors or modulators. Structures of compounds affecting signaling pathways controlling transcription factors regulating HIV‐1 transcription are illustrated. 5HN causes generation of reactive oxygen species and activation of NFκB. AV6 enhances activity of the nuclear factor of activated T‐cells. Disulfiram and 57704 activate Akt signaling. The kinase inhibitor PKC412 promotes phosphorylation of NFκB p65. The benzotriazole derivative HODHBt inhibits STAT5 SUMOylation, promoting nuclear retention. EK‐16A, aplysiatoxin, and debromoaplysiatoxin act as PKC agonists. 5HN, 5‐hydroxynaphthalene‐1,4‐dion; AV6, antiviral 6; HIV, human immunodeficiency virus; HODHBt, 3‐hydroxy‐1,2,3‐benzotriazin‐4(3H)‐one; LRA, latency‐reversing agent; NFκB, nuclear factor κ‐light‐chain‐enhancer of activated B cells; PKC, protein kinase C; STAT5, signal transducer and activator of transcription 5

Reactive oxygen species (ROS), such as hydrogen peroxide (H_2_O_2_), superoxide anions (O_2_^−^), and hydroxyl radicals (OH) cause oxidative stress and induce different biological responses depending on the level of ROS.^108^ High ROS levels cause damage to cellular macromolecules, which can lead to apoptosis or necrosis, while low and intermediate ROS levels can act as second messengers which stimulate antioxidative and inflammatory responses.^108^, ^109^ Specifically, it is known that NFκB becomes activated by intermediate levels of ROS that trigger inflammation.^109^, ^110^, ^111^ Accordingly, reporter genes under control of the HIV‐1 LTR promoter, and HIV‐1 provirus in latently infected Jurkat T cells, are induced in response to treatment with H_2_O_2_, and this effect is dependent upon NFκB binding sites within the HIV‐1 5′ LTR enhancer.^112^ Furthermore, intermediate levels of ROS were shown to activate inhibitor of κB (IκB) kinase and cause degradation of IκB (Figure 1, ROS), in a cell type‐dependent manner, which in T cells may involve the SH2 domain‐containing inositol 5′‐phosphatase 1.^109^ Acetylation of p65 at specific residues enhances NFκB transcriptional activation function,^113^ and, accordingly, it was shown that histone acetyltransferases, such as cyclic AMP response element‐binding protein‐binding protein/p300, may also be involved in ROS‐induced HIV‐1 LTR activation through acetylation of NFκB p65.^114^ Consistent with these observations, the compound 5‐hydroxynaphthalene‐1,4‐dione (5HN), was identified in a HTP LRA screen of natural small molecules, and shown to induce HIV‐1 transcription through ROS generation (Figure 4, Tables 1 and 3).^34^ This compound, found in leaves, roots and bark of the black walnut tree, is a quinone which can be reduced to a semiquinone radical by nicotinamide adenine dinucleotide phosphate oxidoreductase. Under aerobic environmental conditions, semiquinone radicals generate ROS and induce oxidative stress.^115^ Consistent with the observations described above, it was shown that 5HN induces HIV‐1 transcription through ROS‐stimulated NFκB activity (Figure 1, ROS), through a mechanism that is not dependent upon activation of NFAT or protein kinase Cθ (PKCθ).^34^

#### 2.3.2 Antiviral 6

One of the largest HTP screens of small molecules for LRAs to date, involved 200 000 structurally diverse compounds, using primary CD4^+^ T cells infected with a lentivirus expressing secretable alkaline phosphatase (*seap*) and *egfp* reporter genes under control of the HIV‐1 LTR (Table 1).^35^, ^116^ One compound identified in this screen, antiviral 6 (AV6) was found to cause enhanced binding of NFAT (nuclear factor of activated T‐cells) to the viral promoter in J‐Lat cells (clone 9.2) (Figure 4 and Table 3). Notably, this new LRA was shown to cause synergistic induction of HIV‐1 provirus expression in combination with the HDAC inhibitor valproic acid (Table 2). A subsequent study described development of structural analogs with a linear alky linker and HDAC inhibitor functional group attached to the quinoline ring C‐6 position of the parental AV6 structure.^117^ Of these, structures carrying a CONHOH HDAC inhibitor functional group, connected by oxygen to the quinoline ring produced the greatest effect for reversing viral latency. Furthermore, these AV6 analogs were shown to enhance viral transcription mediated through both inhibition of HDAC activity, and stimulation of NFAT DNA binding, but also cause dissociation of positive transcription elongation factor b (pTEFb) from the inhibitory hexamethylene bisacetamide‐induced protein (HEXIM) 7SK small nuclear ribonucleoprotein complex.^117^

#### 2.3.3 Disulfiram/Antabuse; 57704/oxaglaucine

The latency reversing activity of several hybrid polar compounds, including the HDACIs SAHA and hexamethylene bisacetamide was initially shown to be dependent upon the phosphatidylinositol 3‐kinase (PI3K)‐Akt signaling pathway.^118^, ^119^ Subsequent screens for latency reversing activities identified compounds that activate the PI3K‐Akt signaling pathway (Figure 1, PI3K, Akt),^39^, ^72^ including disulfiram, a thiuram disulfide‐containing compound, which was identified as a LRA in a screen of compounds with previously characterized biological activity (Figure 4, Tables 1 and 2). Disulfiram is an FDA‐approved drug prescribed to patients afflicted with alcoholism because it inhibits aldehyde dehydrogenase, leading to increased levels of acetaldehyde, causing an aversive effect that discourages alcohol consumption.^120^ Subsequent to identification as a LRA, disulfiram was also shown to inhibit phosphatase and tensin homology (PTEN), a negative regulator of the Akt signaling pathway (Figure 1, PTEN),^72^ which can account for its effect on reactivation of HIV‐1 transcription.^36^, ^72^, ^121^ Disulfiram is rapidly converted to diethyldithiocarbamic acid in vivo,^122^ and this metabolite was shown to act as a LRA. Because disulfiram had already been in clinical use, it attracted attention for clinical studies aimed at eliminating latent HIV‐1 reservoirs. Although disulfiram administration was shown to induce a transient increase in viremia on its own, no change in the size of latent reservoirs was observed.^123^

A quinoline‐containing compound, designated 57704, was identified as a LRA from a screen of natural products, using HIV‐1 reporter cell lines and CD8^+^‐depleted mononuclear cells isolated from HIV‐1‐infected patient samples (Figure 4, Tables 1 and 3).^39^ Interestingly, the ability of 57704 to activate viral transcription was decreased in cells treated with the PI3K inhibitor wortmannin or the Akt inhibitor IV, and also this compound caused increased phosphorylation of Akt. These observations indicate that it may act as a PI3K‐Akt agonist (Figure 1, Akt), and may specifically target the PI3K p110 isoform α.^90^

#### 2.3.4 PKC412

PKC412 is a derivative of the alkaloid staurosporine, and was identified as a LRA in screens of synthetic and naturally occurring compounds (Figure 4, Tables 1 and 3).^42^ This compound is a broad‐spectrum kinase inhibitor, including for PKC and various protein‐tyrosine kinases,^42^ and has antitumor activity against human myeloma cells, non–small‐cell lung cancer cells, and toward a murine model of myeloproliferative disease.^124^, ^125^, ^126^ PKC412 also induces apoptosis in human multiple myeloma cells, by an effect mediated through Jun N‐terminal kinase activation and upregulation of the transcriptional activator activator protein 1 (AP1).^127^ Efforts investigating the anti‐HIV‐1 latency reversing activity of this compound suggest PKC412 stimulates HIV‐1 transcription by a mechanism involving phosphorylation of NFκB p65,^42^ which suggests that one or more PKC isoforms, or related enzymes, may have inhibitory effects on this pathway (Figure 1).

#### 2.3.5 3‐Hydroxy‐1,2,3‐benzotriazin‐4(3*H*)‐one

The HIV‐1 LTR has three *cis*‐elements for the signal transducer and activator of transcription 5 (STAT5; Figure 1),^128^ which cause activation of transcription in response to γc‐related cytokines (interleukin 2 [IL‐2], IL‐4, IL‐7, and IL‐15). Binding of these cytokines to their corresponding receptors results in tyrosine phosphorylation and subsequent activation of the associated Janus family kinases (JAKs).^129^ IL‐2 predominately activates the JAK1 and JAK3 kinases, which phosphorylate cytoplasmic STAT5, causing translocation to the nucleus where it activates genes mainly involved in proliferation, differentiation and inflammation.^129^ Benzotriazole derivatives, particularly 3‐hydroxy‐1,2,3‐benzotriazin‐4(3*H*)‐one (HODHBt), were shown to reactivate latent HIV‐1 by a mechanism dependent on IL‐2 treatment, which is also required for survival of T_CM (central memory)_ cells in in vitro cultures (Figure 4, Tables 1 and 3).^43^ Using the PASTAA (predicting associated transcription factors from annotated affinities) software program to analyze the differentially expressed genes in HODHBt‐treated cells, the STAT transcription factors were predicted to be affected by this class of compounds for induction of viral transcription.^43^ Accordingly, ChIP analyses from HODHBt‐treated cells indicated recruitment of STAT5A to the HIV‐1 LTR promoter (Figure 1, STAT5), which confirmed direct involvement of STATs in stimulating viral transcription.^43^ However, the benzotriazole derivatives did not induce STAT5 phosphorylation at tyrosine 694 on their own, which is required for activation, but rather cause accumulation of IL‐2‐induced phosphorylated STAT5 by impairing SUMOylation and causing increased levels of phosphorylated STAT5 in the nucleus (Figure 1).^43^ Based on these observations, it might be predicted that benzotriazole compounds may also enhance latency reversing activity of the additional γC‐cytokines (IL‐7, IL‐21, and IL‐15)^130^, ^131^, ^132^ through activation of the STATs.

#### 2.3.6 EK‐16A, aplysiatoxin, and debromoaaplysiatoxin

HIV‐1 transcription is tightly linked to signals generated from the T cell receptor (TCR) in CD4^+^ T cells,^133^, ^134^ which is activated by engagement with major histocompatibility complexes of antigen‐presenting cells.^134^, ^135^ Antigen presentation causes association of p56^lck^, and phosphorylation of the CD4 TCR/CD3 complex, leading to recruitment of ζ‐chain‐associated protein kinase 70 (ZAP‐70 kinase). ZAP‐70 in turn activates phospholipase Cγ, which hydrolyzes phosphatidylinositol (4,5) bisphosphate into the secondary messengers diacylglycerol (DAG) and inositol triphosphate, that stimulate three downstream signaling pathways with different transcriptional activator targets.^100^, ^134^, ^135^, ^136^ In T cells, DAG activates PKCθ and the Ras/Raf/MAPK/ERK‐AP1 pathway.^134^ Because it regulates at least two divergent pathways downstream of the T cell receptor that consequently affect virus expression, PKC represents an important target for modulation by small molecules (Figure 1, PKC).^137^ Accordingly, PKC agonists act as DAG mimetics, and comprise three structural categories, including phorbol esters, cyclic lactones and diterpenes, which include ingenol compounds.^137^ Notably, PKC enzymes have an N‐terminal regulatory domain in which a conserved cysteine‐rich motif, referred to as the C1 domain serves as a docking site for DAG as well as phorbol esters.^138^, ^139^

EK‐16A, an ingenol derivative purified from the root of *Euphorbia kansai*, was identified as a LRA in a screen of compounds from traditional Chinese medicinal herbs, was shown to have more potent activity for reactivation of latent HIV‐1 than prostratin (Figure 4, Tables 1 and 3), and found to predominately activate PKCγ.^52^ Two additional PKC activators aplysiatoxin and debromoaplysiatoxin were identified in a screen of natural compounds purified from marine invertebrates and microorganisms (Figure 4, Tables 1 and 3).^47^ These compounds are produced by blue‐green algae, and were initially identified as agents with proinflammatory and tumor‐promoting activities.^140^ Similar to phorbol esters, these compounds bind the C1 regulatory domain of PKC which facilitates interaction with the cell membrane phospholipid bilayer causing activation of the enzyme.^141^, ^142^, ^143^

### 2.4 Group 4: Transcriptional elongation modulators

#### 2.4.1 Aclacinomycin/aclarubicin

**Figure 5:**
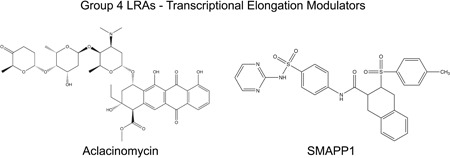
Chemical structures of group 4 LRAs, transcriptional elongation modulators. Illustrated are the chemical structures LRAs identified in screens shown to promote transcriptional elongation from the HIV‐1 LTR. Aclacinomycin causes dissociation of pTEFb from the inhibitory HEXIM1 7SK snRNP complex. SMAPP1 enhances activity of Cdk9 of the pTEFb transcriptional elongation complex. HIV, human immunodeficiency virus; LRA, latency‐reversing agent; SMAPP1, small molecule activator of PP1; snRNP, small nuclear ribonucleoprotein

As indicated above, because any single LRA is likely incapable of inducing a sufficiently broad spectrum of latent provirus, a recent focus for identification of novel activities involves identifying combinations of LRAs that target parallel pathways regulating expression from the provirus genome, with the expectation that this would produce a more robust means of eradicating persistent viral infection. For example, aclacinomycin was identified as a compound that increases the latency‐reversing activity of tumor necrosis factor receptor‐α at low concentrations (Figure 5, Table 1 and 3).^24^ Aclacinomycin, is an FDA‐approved compound which causes cell‐differentiation, and has anticancer activity as a DNA intercalation agent and transcription inhibitor.^144^, ^145^ However, these properties do not seem to contribute to latency reversing activity for HIV‐1, but rather this compound was shown to cause dissociation of the elongation factor pTEFb from its inhibitory subunit HEXIM1, and this effect can account for its effect on enhancing viral transcription (Figure 1, pTEFb).^24^

#### 2.4.2 Small molecule activator of protein phosphatase 1

Protein phosphatase‐1 (PP1) was shown to modulate HIV‐1 transcription by interaction with the viral transactivator TAT, which promotes translocation of PP1 to the nucleus where it indirectly enhances viral transcription by increasing catalytic activity of the pTEFb CDK9 subunit^146^ (Figure 1, pTEFb). Accordingly, expression of a short PP1‐binding peptide derived from the nuclear inhibitor of PP1 (cdNIPP1) disrupts the TAT–PP1 interaction, causing alteration of CDK9 (cyclin‐dependent kinase 9) phosphorylation which inhibits viral transcriptional elongation.^147^ On the basis these observations a panel of small molecules targeting a PP1 noncatalytic subunit was evaluated for effects on both the inhibition and activation of viral transcription,^40^, ^148^, ^149^ and in these efforts compounds with a sulfonamide linker were found to be particularly effective for inducing viral transcription.^40^ This observation led to development of a compound library of sulfonamide‐containing small molecules, from which a novel compound, designated small molecule activator of PP1 (Figure 1, small molecule activator of protein phosphatase 1 [SMAPP1]), was identified as a potent stimulator of HIV‐1 transcription in latently infected primary T cells (Figure 5, Tables 1 and 3).^40^ Accordingly, SMAPP1 was shown to enhance phosphorylation of CDK9 at the regulatory Thr186 (Threonine 186) phosphorylation site (Figure 1).^40^ Subsequent structural analysis demonstrated direct interaction of SMAPP1 with the PP1 C‐terminal groove. This compound was also subsequently combined with a nanoparticle delivery system to increase in vivo bioavailability and enhance activity toward latent provirus.^150^

## 3 CHEMICAL BIOLOGY OF HIV PROVIRUS LRAS

Identification of novel compounds with HIV latency reversing activity is a relatively young endeavor; we describe results from 18 small molecule screens, but only two of which have involved extensive unbiased libraries of 100K compounds or more (Table 1).^28^, ^35^ Considering that 100s of HTP screens involving very large libraries have been focused toward identification of novel cancer drugs over the past 20 years,^151^, ^152^ efforts toward identification of novel HIV LRAs by comparable strategies appear to be in their infancy. Furthermore, most drugs targeting cancer biology, initially identified in HTP small molecule screens, have typically undergone multiple rounds of medicinal chemistry optimization, subsequent to SAR analysis.^151^, ^152^ However, to date only a few LRA compounds have been subjected to SAR studies to produce optimized activities.^33^, ^117^, ^153^ Therefore, we suggest that SAR of novel LRAs, and implementation of additional medicinal chemistry tools, such as scaffold hopping,^154^ fragment‐based drug design,^155^ Lipinski's rule of five,^156^ and analog‐based drug design,^157^ will also contribute to identification of novel effective agents for reactivation of latent HIV provirus.

The HIV‐1 5′ LTR is, arguably, among the most thoroughly characterized promoters in human cells, and in this respect, it is interesting that novel LRAs identified in unbiased HTP small molecule screens were found to affect well‐characterized mechanisms for regulation of HIV‐1 transcription, including PKC‐NFκB signaling, histone deacetylases, and regulation of transcriptional elongation, but also previously less well‐characterized mechanisms that have received less prior attention. For example, novel LRAs have illustrated the role of JAK‐STAT signaling, and noncanonical NFκB signaling initiated by ROS and PTEN‐Akt for regulation of HIV transcription.^43^, ^158^, ^159^, ^160^ Furthermore, the effect of novel LRAs may illuminate previously unrecognized regulatory mechanisms, for example understanding the effect of PKC412 for reactivation of LTR expression,^42^ will likely reveal novel mechanisms for regulation of NFκB function. Considering the complexity and combination of mechanisms that control HIV expression, we expect that many additional novel mechanisms will be revealed by results from further large scale HTP small molecule library screens.

## 4 LRAS WITH A COMMON PRIVILEGED STRUCTURE AFFECT VARIOUS TARGETS

**Figure 6:**
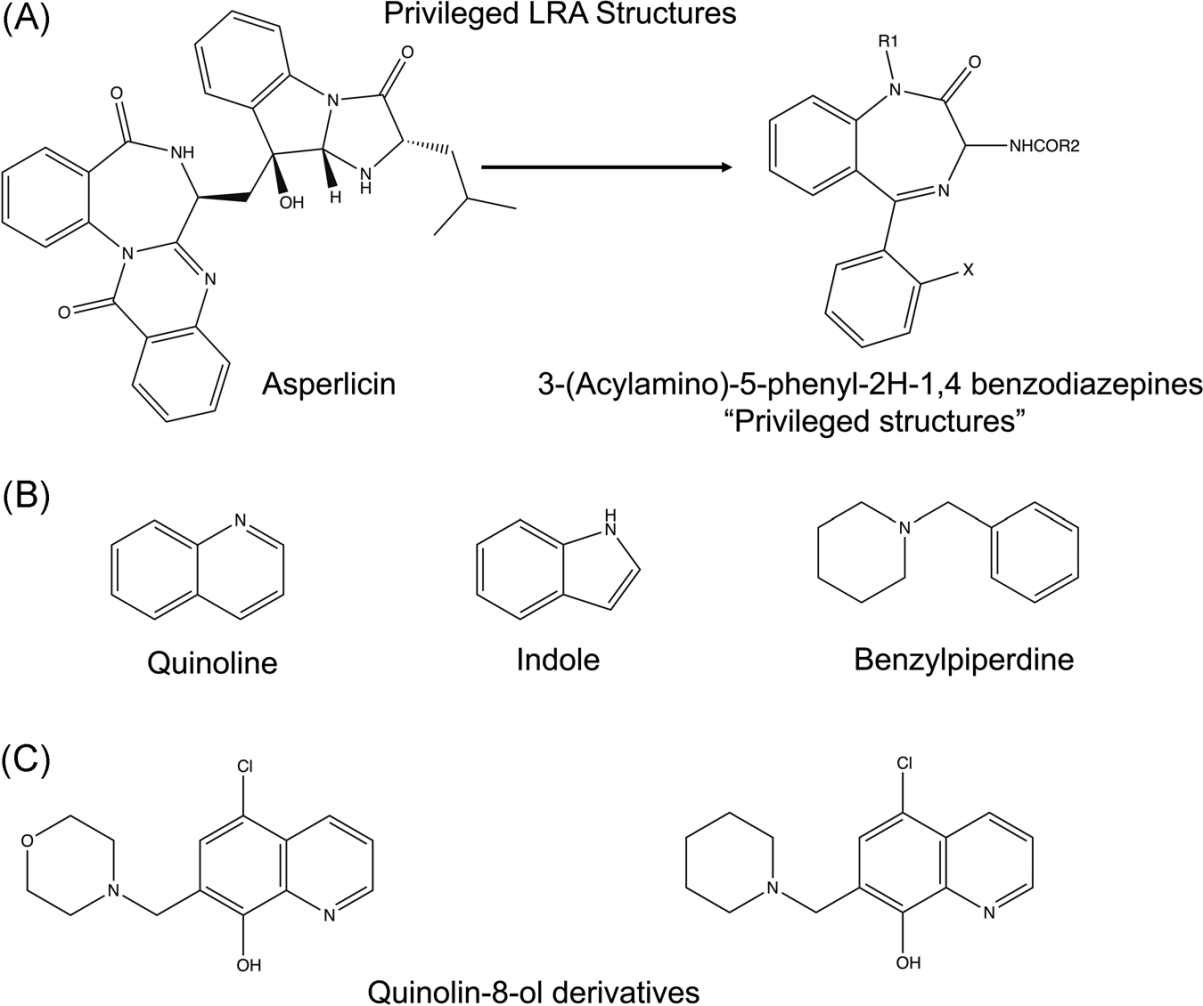
Privileged chemical structures relating to HIV‐1 latency reversing activities. Therapeutic agents recognizing receptors that bind the natural product asperlicin (A) include compounds related to CCK, 3‐(acylamino)‐5‐phenyl‐2*H*‐1,4‐benzodiazepines. Derivatives of this core structure exhibit affinity toward diverse biochemical targets. Screens for latency reversing agents have identified three examples of chemical scaffolds (B) that may represent privileged structures for novel drug discovery. Chemical structures of two HIV‐1 latency reversing agents derived from the privileged quinolin‐8‐ol structure are illustrated (C). CCK, cholecystokinin; HIV, human immunodeficiency virus; LRA, latency‐reversing agent

The LRAs MMQO, AV6, 57704, and PH03 were identified in four separate screens, but carry a common central quinoline structure,^28^, ^35^, ^38^, ^39^ which we propose may represent a privileged structural motif. In chemical biology and medicinal chemistry, privileged structures are represented by a semirigid molecular scaffold that when modified by multiple hydrophobic residues or functional groups produce versatile binding properties that confer the capacity to interact with diverse biochemical targets.^161^, ^162^ The term “privileged structure” was initially coined by Evans in 1988, to describe affinity of asperlicin derivatives toward a diverse collection of receptors (Figure 6A).^163^ This term has subsequently been used frequently, but there are no strict rules defining particular structures as “privileged.”^164^, ^165^ Typically, they carry either two or three rings connected through a ring‐fusion or single bonds. Figure 6B illustrates examples of common privileged structures found in drugs and natural products. For discovery of novel LRAs, derivatives of the related privileged scaffold quinolin‐8‐ol, were screened for more potent latency reversing activity, which revealed that compounds carrying either a morpholine or piperdine ring at position 7 and a chloro group at position 5 of the quinoline ring, induced viral transcription at relatively lower concentrations than the parental compound (Figure 6C).^37^

The four quinoline‐containing LRAs identified in small molecule screens have various biological targets. MMQO activates HIV‐1 transcription through inhibition of the BET protein BRD4.^59^ BET inhibitors, typified by the compound JQ1, had previously been used as anti‐inflammatory and anticancer agents, but later several groups demonstrated HIV‐1 latency reversing activity for JQ1 and compounds with related structures through inhibition of BRD4 (Figure 1, BETi).^166^, ^167^, ^168^ MMQO has significant structural differences to previously characterized BET inhibitors,^59^ and unlike JQ1, HIV‐1 latency reversing activity of MMQO is TAT‐independent (Figure 1, Tat), which suggests that BRD4 is likely involved in additional mechanisms repressing viral transcription than competing for binding of TAT with pTEFb. Two additional structurally divergent pteridine‐containing compounds identified as LRAs, BI‐2536 and BI‐6727,^46^ also have BD inhibitory function, but the precise mechanism through which they stimulate viral transcription has not been elucidated.

The additional quinolone‐containing compounds identified as LRAs appear to have additional distinct targets. Compound 57704 (Figure 4) acts as a LRA through activation of PI3K and PI3K‐Akt signaling (Figure 1, Akt),^39^ but through a different mechanism than disulfiram, which has a completely different structure (Figure 1). AV6 causes activation of LTR transcription through NFAT, but again, by a mechanism that has not been determined. The fourth quinoline‐containing LRA, designated PH03,^28^ has previously been examined as a potential probe for in vivo imaging of tau pathology in Alzheimer's disease.^169^, ^170^, ^171^ This compound, also designated BF‐170, seems to affect diverse molecular functions as it produced activity in a variety of additional screens, including for agonists of steroid receptors, enhancers of survival of motor neuron 2 expression, inhibitors of Marburg virus entry, inhibitors of human muscle pyruvate kinase, and inhibitors of the histone lysine methyltransferase G9a.^172^, ^173^, ^174^, ^175^, ^176^, ^177^, ^178^, ^179^, ^180^, ^181^, ^182^, ^183^, ^184^, ^185^ However, whether any of these effects account for its latency reversing activity for HIV‐1 provirus has not yet been elucidated.

## 5 PROSPECTIVE FOR FUTURE LRA IDENTIFICATION AND DEVELOPMENT

Critical features for effective LRAs include a capacity to broadly reactivate viral transcription, independent of the chromosomal integration site, and without causing adverse effects on cellular homeostasis, T cell activation or toxicity. With this consideration, some currently well studied LRAs, including PKC agonists and nonclass HDACIs may not be ideal for this purpose. PKC agonists typically cause broad‐scale cytokine production,^186^, ^187^ and nonclass HDACIs can produce global alterations in transcription,^188^, ^189^ that may cause significant toxic effects. Consequently, the development of LRAs that target unique, and currently less‐characterized, mechanisms that modulate HIV‐1 provirus transcription may be a priority for future investigations. As outlined, currently characterized LRAs function through diverse mechanisms of action (Figure 1), all of which also affect cellular transcriptional regulatory mechanisms. An ideal LRA for HIV “shock and kill” therapy would specifically reactivate HIV‐1 transcription without causing alterations of cellular genes; a transcriptional activator that specifically regulates HIV‐1 transcription would represent an ideal target for this purpose. HIV‐1 does encodes its own transactivator TAT (Figure 1, Tat), but which functions by binding the nascent TAR RNA to activate elongation of paused RNA Polymerase II complexes.^190^, ^191^ Several LRAs function to indirectly enhance TAT activity by upregulating function of Cdk9/cyclin T of the pTEFb elongation complex^24^, ^40^, ^192^, ^193^ (Figure 1, pTEFb); however, these factors also regulate numerous cellular genes, and consequently compounds affecting this mechanism do not specifically target HIV‐1 transcription. Consequently, development of agents capable of enhancing TAT function, or inhibiting factors that negatively regulate TAT, would provide a highly specific means of enhancing provirus expression. Latently infected cells harboring transcriptionally silent provirus theoretically would not express TAT protein.^190^ However, the fact that BET inhibitors, and Cdk9 agonists (Figure 1, BETi, Cdk9), induce HIV‐1 provirus expression supports the contention that latent HIV‐1 must produce sporadic transcripts to maintain low levels of virus gene products in these cells.^24^, ^192^, ^193^ In this context, perhaps the most effective combination of LRAs, to produce an effective “shock,” would include agents that elevate basal HIV‐1 provirus transcription, plus simultaneously stimulating activity of HIV‐1 TAT protein.

An alternative strategy toward specifically reactivating HIV expression, relative to global transcription, would involve stimulating combinations of factors that would not affect most cellular genes but which would cause induction of expression from the HIV‐1 LTR. This may represent an “Achilles heel” of the HIV‐1 provirus, in that the 5′ regulatory region contains binding sites for more than 30 different sequence‐specific transcriptional regulatory factors.^100^, ^194^, ^195^ Effects on combinations of factors that bind the LTR typically produce synergistic effects on provirus reactivation.^100^, ^194^, ^195^ Consequently, it may be productive to implement small molecule screens to specifically identify compounds that are effective for provirus reactivation in combination with previously FDA approved LRAs. Additionally, we note that most of the small molecule screens for novel LRAs (Table 1), except for two small scale studies,^33^, ^46^ were performed using T‐cell lines or primary cells, and there has not yet been significant effort toward identifying LRAs that may be specifically effective in monocyte‐macrophage lineages. Because these cell types represent important reservoirs for virus in patients on ART,^196^, ^197^, ^198^ it may be necessary to target unique aspects of provirus regulation in these cells.

## 6 CONCLUDING REMARKS

While current antiretroviral therapies effectively control HIV‐1 replication and productive viral infection, they do not affect latently infected cells bearing transcriptionally repressed provirus,^1^, ^2^, ^3^, ^4^ and development of a therapeutic means to eliminate these latent reservoirs is necessary for long‐term management of the HIV‐1 epidemic. Therapies devised to specifically target the latently infected population will require a means of distinguishing these cells from their uninfected counterparts. Considerable research effort has been directed at identifying biomarkers of cells latently infected with HIV‐1 but to date no suitable macromolecules have been identified. Currently, the only known means of exposing the latently infected population is to force reactivation of HIV‐1 expression to produce viral proteins that mark these cells as infected. This reality has driven efforts to identify novel LRAs with more potent and globally encompassing effects. Results from preclinical and clinical analysis of FDA approved LRAs has revealed a need for optimization of compounds with this activity to produce sufficiently effective responses to enable elimination of the latently infected population.^199^, ^200^ Importantly however, all of the clinical trials performed to date have involved drugs that had been repurposed from treatment of other conditions^16^, ^201^; and consequently, the full capability of LRAs with respect to the “shock and kill” strategy in general may not be realized until trials with compounds specifically designed and optimized for this purpose. Furthermore, application of recent developments in drug delivery involving nanocarriers, such as polymeric nanoparticles, liposomes, lipid nanoparticles, and dendrimers, to encapsulate LRAs,^202^, ^203^, ^204^ could increase their circulation or tissue retention time, solubility and bioavailability, enhance drug potency, and reduce cellular toxicity^205^ and thereby improve overall success of this approach.

In addition to improving effectiveness of LRAs, success of the proposed shock and kill strategy will require combined intervention to improve killing of cells where virus replication has been reactivated.^19^, ^20^, ^206^, ^207^, ^208^ The failure of LRAs to reduce the latently infected population in initial clinical trials is likely a consequence of multiple factors, including that resting memory CD4^+^ T cells express elevated levels of antiapoptotic molecules such as Bcl‐2,^209^ which may interfere with virus‐induced cell death. Additionally, some LRAs used in these trials have negative effects on CD8^+^ T cell function and cause upregulation of T‐cell exhaustion markers on latently infected cells which reduces response to cell mediated immunity.^210^, ^211^, ^212^ Additionally, the majority of latent provirus in patients on ART were shown to express *gag* escape mutations that cause insensitivity to the cytotoxic T lymphocyte response.^213^ Additional interventions to overcome these barriers may include passive immunotherapy, therapeutic vaccination, or manipulation of apoptosis regulatory pathways.^214^, ^215^

Finally, a variety of additional strategies to purge HIV‐1 infection have been proposed and are currently under development, including the use of broadly neutralizing monoclonal antibodies, T‐cell immunotherapy, chimeric antigen receptor T cell therapy, gene editing, immune cell depletion and transplantation, as well as strategies intended to prevent reactivation of HIV‐1 expression, referred to as “lock and block.”^216^, ^217^ Because of the extent that HIV‐1 infection is thought to penetrate multiple cell types and tissue compartments,^218^, ^219^, ^220^, ^221^, ^222^ it seems likely that no single strategy will provide a “cure all” therapy, and combinations of various treatments may be necessary for routine curative intervention.
